# Robotic vs. bedside staplers in robotic lung resection: a real-world cohort study

**DOI:** 10.1007/s11701-026-03772-0

**Published:** 2026-08-06

**Authors:** Brian Mitzman, Yu-Ting Chi, Peng-Lin Lin, I-Fan Shih, Feibi Zheng

**Affiliations:** 1 Division of Cardiothoracic Surgery, Department of Surgery, University of Utah Health, Salt Lake City, UT USA; 2 Global Health Economics & Outcomes Research, Intuitive, Sunnyvale, CA USA; 3 Michael E. DeBakey Department of Surgery, Baylor College of Medicine, Houston, TX USA; 4 Huntsman Cancer Institute, 1950 Circle of Hope, Salt Lake City, 84112 UT USA

**Keywords:** Lobectomy, Segmentectomy, Wedge resection, Robotic stapler, Bedside stapler

## Abstract

Comparative studies of stapler strategy within robotic lung resection remain limited, particularly beyond lobectomy and with contemporary staplers. This study evaluated clinical and economic outcomes associated with robotic versus bedside staplers in robotic lung resections. We conducted a retrospective cohort study using the Premier Healthcare Database (2019–2023), including adults who underwent robotic lobectomy or sublobar resection. Patients were classified into robotic stapler and bedside stapler groups. Overlap weighting (OW), a propensity score weighting method targeting the average treatment effect in the overlap population (ATO), was applied to balance measured baseline characteristics. The prespecified primary outcome was postoperative air leak. Secondary outcomes included other postoperative complications, conversion to thoracotomy, operative time, length of stay (LOS), and hospital costs during the index hospitalization and 30-day perioperative period. Among 15,140 patients, robotic staplers were associated with lower air leak (10.9% vs. 15.2%), pneumothorax (8.5% vs. 11.4%), and pneumonia (1.1% vs. 2.8%), as well as lower conversion (0.4% vs. 4.7%), transfusion (1.0% vs. 1.8%), shorter operative time (209 vs. 225 min), and length of stay (3.8 vs. 5.4 days) (all *p* < 0.05). Index hospitalization cost was lower with robotic staplers (USD 25,887 vs. 27,432; Δ − 1,545), with similar findings for 30-day perioperative cost. In the sensitivity analysis including mixed-use cases, associations remained directionally consistent, including lower air leak (11.3% vs. 15.3%), conversion (0.8% vs. 4.6%), and total hospital cost (USD 26,370 vs. 27,365). In robotic lung resections, robotic staplers were associated with fewer complications and conversions, shorter operative time and LOS, and lower index and 30-day hospital costs. These findings were consistent in a sensitivity analysis including mixed-use cases but should be interpreted cautiously because residual confounding cannot be excluded.

**Supplementary Information:**

The online version contains supplementary material available at 10.1007/s11701-026-03772-0.

## Introduction

Lung cancer is the leading cause of cancer mortality worldwide [1]. Surgical resection remains the primary treatment for early-stage disease, and is most commonly performed as lobectomy, segmentectomy, or wedge resection. Robotic-assisted thoracic surgery (RATS) has seen increasing adoption over video-assisted thoracic surgery (VATS), driven by evidence of more precise lymph node dissection, and faster recovery [2, 3]. At the same time, stapling technology has evolved from manual and powered bedside staplers to console-integrated robotic staplers that enable surgeon-controlled firing and articulation [4–8].

Despite these advances, many robotic surgeons opt for bedside stapling and the comparative effectiveness of robotic staplers versus bedside staplers remains uncertain. Most studies focus on RATS versus VATS rather than stapler modality [9, 10]. Among stapler-focused studies, results have been inconsistent, and evidence for segmentectomy or wedge resection is scarce [11–13]. Economic data are particularly limited and were derived from older stapler generations, small cohorts, or heterogeneous bedside comparators.

To address these gaps, we compared clinical and economic outcomes of robotic versus bedside staplers in robotic lung resections, using a large, nationally representative U.S. hospital database. This analysis followed an a priori protocol that was finalized, peer-reviewed, and published before data extraction [14].

## Materials and methods

### Study design

This retrospective cohort study used data from Premier Healthcare Database (PHD). PHD is a comprehensive U.S. hospital-based, HIPAA-compliant database containing detailed inpatient and outpatient billing and clinical information from geographically diverse hospitals [15], capturing approximately 25% of the annual inpatient admissions in the United States [16]. The study included adult patients (≥ 18 years) who underwent elective, fully robotic-assisted lobectomy, segmentectomy, or wedge resection between January 1, 2019, and December 31, 2023.

### Study population

Patients were identified using the ICD-10-PCS and CPT codes for robotic lung resections (Supplementary Materials) [17]. Procedures were classified as lobectomy or sublobar resection (segmentectomy/wedge), as ICD-10-PCS does not reliably distinguish between segmentectomy and wedge resection. If multiple procedure codes were recorded during the same encounter, patients were classified as lobectomy. Only the first robotic lung resection per patient was included. Patients with missing baseline characteristics were excluded.

### Stapler exposure

Patients were categorized into robotic stapler or bedside stapler groups. The robotic stapler group included cases using Intuitive SureForm staplers without any bedside staplers, whereas the bedside stapler group included cases without robotic stapler use. Stapler modality was identified using a prespecified chargemaster text-based algorithm defined a priori [14]. Cases in which both SureForm and bedside staplers were identified were excluded from the primary analysis to reduce exposure heterogeneity. Chargemaster data do not capture the sequence of stapler use or the clinical rationale for using multiple stapler modalities within the same procedure. Mixed-use cases may represent more complex procedures or cases requiring intraoperative changes in surgical approach. Consistent with the published protocol, these mixed-use cases were included in the robotic stapler group in a sensitivity analysis evaluating the larger cohort.

### Outcomes

The prespecified primary outcome was postoperative air leak. Secondary clinical outcomes included other postoperative complications, transfusion during the index hospitalization, conversion to open thoracotomy, operative time, ICU utilization, and length of stay (LOS). Complications included bleeding, bronchopleural fistula, pneumonia, acute post-hemorrhagic anemia, pneumothorax, and infection and were identified using ICD-10-CM/PCS and CPT codes. Air leak was identified from coded postoperative air leak events during the index hospitalization and did not specifically distinguish prolonged air leak. Economic outcomes included total hospital cost during the index hospitalization and total 30-day perioperative cost from the hospital perspective, winsorized at the 1st and 99th percentiles [18–21]. This was not a formal cost-effectiveness analysis.

### Baseline characteristics

Baseline covariates were prespecified based on clinical relevance and the published protocol and included age, sex, race, payor type, COPD/emphysema, Charlson Comorbidity Index (CCI), obesity, malignancy status, teaching hospital status, hospital bed size, urban or rural location, thoracic surgeon status, procedure type, and physician and hospital robotic lung resection volumes. Volumes were defined as the total number of robotic lung resections performed during the study period and were analyzed as continuous variables descriptively and dichotomized at the median in exploratory multivariable models.

### Statistical analysis

Overlap weighting (OW) was used as a propensity score weighting method [22]. The propensity score was estimated using logistic regression including all prespecified baseline covariates listed above, with standard mean difference (SMD) values < 0.10 indicating adequate balance. OW was selected instead of nearest-neighbor matching because OW retains contributions from all observations rather than discarding unmatched cases, and instead of standard inverse probability of treatment weighting because extreme propensity scores can generate very large inverse-probability weights. OW targets the ATO, representing patients for whom either stapler strategy was reasonably plausible based on measured characteristics.

Baseline characteristics were summarized using frequencies (percentages) for categorical variables, means (standard deviation) for length of stay, and medians (interquartile range) for operative time. Group comparisons were performed using chi-squared or Fisher’s exact tests for categorical variables and Welch’s t-test or Mann–Whitney U test for continuous variables, as appropriate.

Multivariable logistic regression was used to identify risk factors for air leak and conversion, within the overlap-weighted cohort. Candidate variables were drawn from the prespecified clinical and institutional covariates, and variables associated in univariable analyses were retained in the final models. These models were intended for risk-factor exploration rather than to re-estimate the primary stapler-group effect. Subgroup analyses were performed for lobectomy and sublobar resection.

All tests were two-sided, with *p* < 0.05 considered statistically significant. Analyses were conducted using R software (version 4.4.1) [23].

### Ethics

This study used retrospective, fully de-identified data from PHD, and was exempt from IRB review; no informed consent was required.

## Results

### Baseline characteristics

**Fig. 1:**
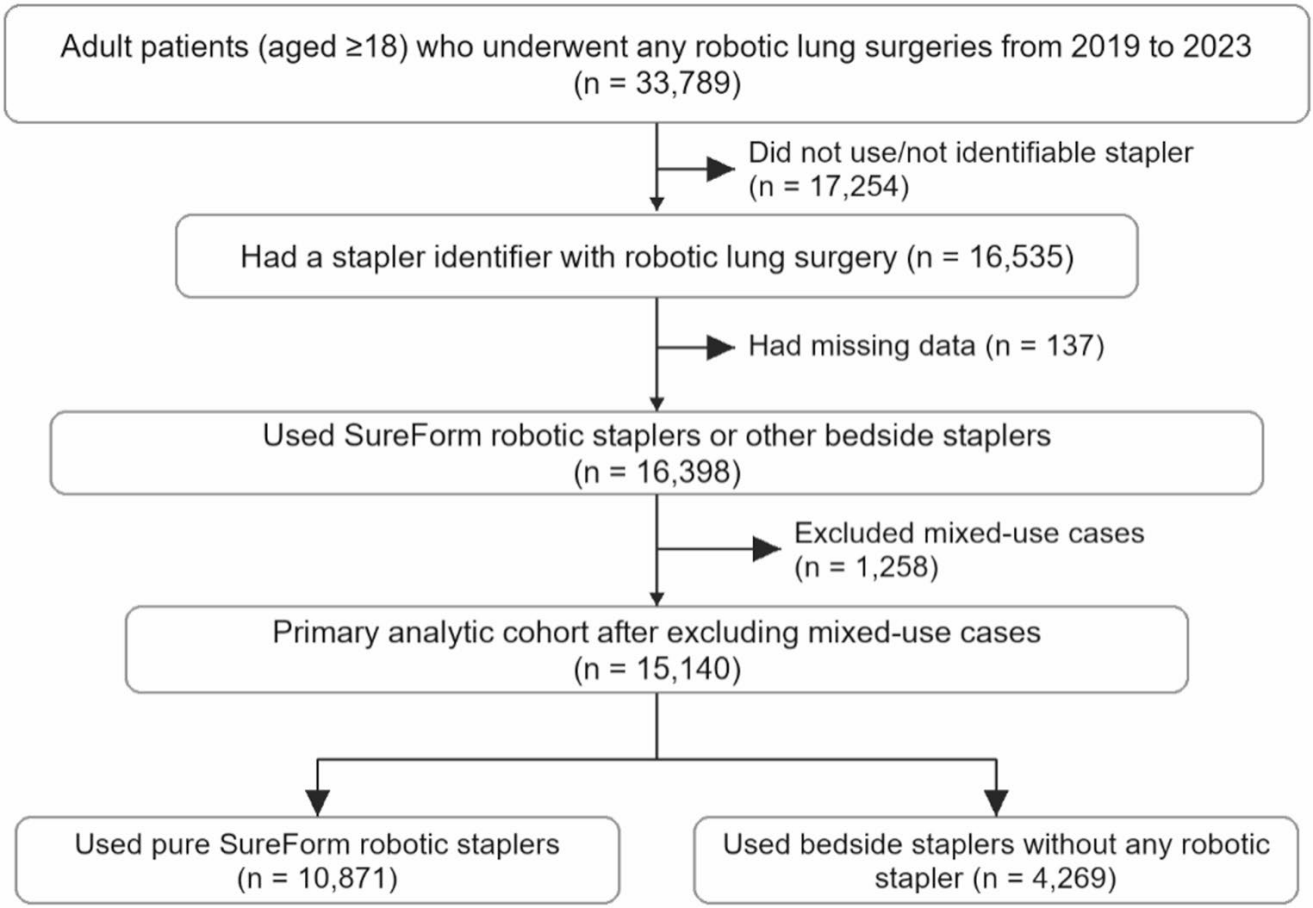
Study flow chart

A total of 15,140 patients were included (Fig. 1), with 10,871 (71.80%) patients in the robotic stapler group and 4269 (28.20%) patients in the bedside stapler group. After OW, the effective sample size was 10,856 (robotic: 7825; bedside: 3031), and all measured covariates were balanced (Table 1).

**Table 1:** Baseline characteristics by stapler type in robotic lung resection before and after overlap weighting

Characteristics	Summary statistics		*p*-value	ASMD	Summary statistics		*p*-value	ASMD
	Before weighting				After weighting			
	Robotic stapler	Bedside stapler			Robotic stapler	Bedside stapler		
	(*N* = 10871)	(*N* = 4269)			**(** ***N*** * = 7825)	**(** ***N*** * = 3031)		
*Age: Mean (SD)*	66.33 (10.96)	67.5 (11.7)	< 0.001	0.1032	66 (11.15)	66 (11.87)	1	< 0.001
***Gender***			< 0.001				1	
Female	6182 (56.87%)	2250 (52.71%)		0.0837	54.01%	54.01%		< 0.001
Male	4689 (43.13%)	2019 (47.29%)		0.0837	45.99%	45.99%		< 0.001
***Race Group***			< 0.001				1	
Black	936 (8.61%)	503 (11.78%)		0.105	12.05%	12.06%		< 0.001
Other/unknown	1246 (11.46%)	433 (10.14%)		0.0425	10.80%	10.79%		< 0.001
White	8689 (79.93%)	3333 (78.07%)		0.0455	77.15%	77.15%		< 0.001
***Payor***			< 0.001				1	
Commercial	2877 (26.46%)	948 (22.21%)		0.0994	26.56%	26.56%		< 0.001
Medicaid	734 (6.75%)	270 (6.32%)		0.0173	6.98%	6.99%		< 0.001
Medicare	6785 (62.41%)	2934 (68.73%)		0.1332	62.76%	62.75%		< 0.001
Other	475 (4.37%)	117 (2.74%)		0.088	3.74%	3.75%		< 0.001
***COPD/emphysema***			0.052				1	
NO	6618 (60.88%)	2525 (59.15%)		0.0353	59.57%	59.55%		< 0.001
YES	4253 (39.12%)	1744 (40.85%)		0.0353	40.43%	40.45%		< 0.001
***CCI***			< 0.001				1	
CCI < 1	4072 (37.46%)	1408 (32.98%)		0.0938	34.66%	34.64%		< 0.001
CCI ≥ 1	6799 (62.54%)	2861 (67.02%)		0.0938	65.34%	65.36%		< 0.001
***Obesity***			< 0.001				1	
No obesity	9138 (84.06%)	3723 (87.21%)		0.0899	85.27%	85.25%		< 0.001
BMI 30–39.9	1274 (11.72%)	413 (9.67%)		0.0662	10.91%	10.92%		< 0.001
BMI ≥ 40	459 (4.22%)	133 (3.12%)		0.0589	3.82%	3.83%		< 0.001
***Teaching Hospital***			< 0.001				1	
NO	3933 (36.18%)	433 (10.14%)		0.6488	17.92%	17.91%		< 0.001
YES	6938 (63.82%)	3836 (89.86%)		0.6488	82.08%	82.09%		< 0.001
***Hospital Bed Size***			< 0.001				1	
<300	2012 (18.51%)	443 (10.38%)		0.2329	13.39%	13.39%		< 0.001
300–399	1016 (9.35%)	406 (9.51%)		0.0056	10.57%	10.57%		< 0.001
400–499	1819 (16.73%)	262 (6.14%)		0.3377	10.76%	10.76%		< 0.001
≥ 500	6024 (55.41%)	3158 (73.98%)		0.3959	65.28%	65.28%		< 0.001
***Urban/Rural***			< 0.001				1	
Rural	432 (3.97%)	30 (0.7%)		0.2177	1.35%	1.35%		< 0.001
Urban	10,439 (96.03%)	4239 (99.3%)		0.2177	98.65%	98.65%		< 0.001
***Performed by Specialist***			0.002				1.000	
NO	4289 (39.45%)	1566 (36.68%)		0.0571	43.48%	43.48%		< 0.001
YES	6582 (60.55%)	2703 (63.32%)		0.0571	56.52%	56.52%		< 0.001
**Hospital Volume**: Mean (SD)	401.07 (311.22)	956 (858.4)	< 0.001	0.8595	488.5 (332.62)	488.5 (528.61)	1	< 0.001
**Physician Volume**: Mean (SD)	196.97 (187.8)	594.3 (651.74)	< 0.001	0.8285	225.19 (186.53)	225.19 (253.84)	1	< 0.001
***Procedure Name***			< 0.001				1	
Lobectomy	5847 (53.79%)	2589 (60.65%)		0.139	55.87%	55.85%		< 0.001
Segmentectomy/wedge resection	5024 (46.21%)	1680 (39.35%)		0.139	44.14%	44.12%		< 0.001
***Malignant Cancer***			< 0.001				1	
NO	3446 (31.7%)	1515 (35.49%)		0.0803	33.5%	33.51%		< 0.001
YES	7425 (68.3%)	2754 (64.51%)		0.0803	66.5%	66.49%		< 0.001

ASMD: absolute standardized mean difference; N*: Effective sample size after weighting; SD: standard deviation; BMI: body mass index; CCI: Charlson comorbidity index

### Clinical outcomes

After OW, robotic staplers were associated with lower rates of overall complications (19.75% vs. 26.25%), conversion (0.35% vs. 4.65%), transfusion (1.04% vs. 1.79%), and shorter LOS (3.8 vs. 5.4 days; Δ − 1.6 days) compared with bedside staplers (all *p* < 0.05). Rates of air leak (10.91% vs. 15.19%), pneumothorax (8.48% vs. 11.44%), and pneumonia (1.09% vs. 2.84%) were also lower, and operative time was shorter (209 vs. 225 min) (all *p* < 0.05) (Table 2).

**Table 2:** Clinical outcomes using pure robotic stapler cohort before and after overlap weighting

Clinical Outcomes	Summary statistics		*p*-value	Summary statistics		*p*-value
	Before weighting			After weighting		
	Pure robotic stapler (*N* = 10871)	Bedside stapler (*N* = 4269)		Robotic stapler (N* = 7825)	Bedside stapler (N* = 3031)	
***Overall Complications***	2386 (21.95%)	1060 (24.83%)	< 0.001	19.75%	26.25%	< 0.001
Air Leak	1313 (12.08%)	616 (14.43%)	< 0.001	10.91%	15.19%	< 0.001
Pneumothorax	1034 (9.51%)	465 (10.89%)	0.011	8.48%	11.44%	< 0.001
Bronchopleural Fistula	16 (0.15%)	17 (0.4%)	0.006	0.16%	0.23%	0.462
Bleeding	480 (4.42%)	181 (4.24%)	0.659	3.95%	4.82%	0.048
Acute Post Hemorrhagic Anemia	471 (4.33%)	175 (4.1%)	0.561	3.91%	4.65%	0.085
Pneumonia	148 (1.36%)	98 (2.3%)	< 0.001	1.09%	2.84%	< 0.001
Infection	33 (0.30%)	19 (0.45%)	0.216	0.31%	0.58%	0.038
***Transfusion***	113 (1.04%)	83 (1.94%)	< 0.001	1.04%	1.79%	0.003
***Conversion***	45 (0.41%)	139 (3.26%)	< 0.001	0.35%	4.65%	< 0.001
***Operative Time***: *Median (IQR)*	210 (150–270)	206 (157–270)	0.850	209 (150–266)	225 (165–295)	< 0.001
***Length of Stay***,*** LOS***: *Mean (SD)*	3.8 (4.4)	4.9 (5.5)	< 0.001	3.8 (4.8)	5.4 (5.9)	< 0.001
***ICU***	1985 (18.26%)	2340 (54.81%)	< 0.001	19.34%	38.63%	< 0.001

Data presented as frequency (%), mean (SD), or median (IQR). N*: Effective sample size after weighting

### Costs

After OW, index hospitalization costs were lower with robotic staplers than with bedside staplers (USD 25,887.3 vs. 27,432.2; *p* < 0.001; Δ − 1544.9 USD). Robotic stapler cases had lower room and board, operating room, and respiratory therapy, whereas stapler and anesthesia cost were higher. Total 30-day perioperative cost were also lower with robotic stapler group (USD 27,546.3 vs. 29,233.6; *p* < 0.001; Δ − 1687.3 USD) (Table 3).

**Table 3:** Costs Using Pure Robotic Stapler Cohort Before and After Overlap Weighting

Costs items	Summary statistics		*p*-value	Summary statistics		*p*-value
Mean (SD)	Before weighting			After weighting		
	Pure Robotic stapler (*N* = 10,871)	Bedside stapler (*N* = 4269)		Robotic stapler (N* = 7825)	Bedside stapler (N* = 3031)	
*Total hospital costs*	26,039.7 (14,350.2)	24,743 (15,029.1)	< 0.001	25,887.3 (14,548.3)	27,432.2 (16,911.6)	< 0.001
Stapler	2,600.8 (2,456.2)	1,245.6 (1,279.6)	< 0.001	2,577.5 (2,436)	1,392.8 (1,515.5)	< 0.001
Other Surgical Supply	3,611.7 (3,715.9)	3,759.1 (2,968.6)	0.168	3,666.7 (3,816)	3,377.6 (3,132)	< 0.001
Room and Board	5,758.1 (6,166.4)	5,762.8 (6,816.4)	0.03	5,678.8 (6,214.3)	7,239.9 (7,681.9)	< 0.001
Anesthesia	752.7 (769.8)	717 (596.5)	< 0.001	698.1 (701.3)	679.8 (692.9)	0.230
Operating Room†	7,969.3 (4,722.1)	7,409.7 (4,214)	0.009	7,798.6 (4,603.2)	7,815.9 (4,620.8)	0.864
Recovery Room	713.3 (558)	638.1 (594.4)	< 0.001	728.8 (552.7)	662.3 (663.1)	0.423
Pharmacy	1,114.3 (964.5)	1,164 (1,190.1)	0.248	1,132.9 (1,010.4)	1,360.4 (1,287.7)	< 0.001
Respiratory Therapy	421.7 (801.7)	598.2 (1,601)	0.002	436.7 (814.6)	612.3 (1,129.4)	< 0.001
Miscellaneous	2,553.7 (2,159.9)	3,265.1 (2,807.9)	< 0.001	2,644.4 (2,253.5)	3,377.6 (3,132)	< 0.001
***Total 30-day perioperative costs***	27,652 (16,354)	26,476 (17,268.2)	< 0.001	27,546.3 (16,606.3)	29,233.6 (19,296.8)	< 0.001

Data presented as mean (SD). N*: Effective sample size after weighting

^†^Operating room cost excludes stapler cost

### Sensitivity analysis

The sensitivity analysis included the 1258 mixed-use cases in the robotic stapler group, yielding 12,129 robotic stapler cases and 4269 bedside-only cases. After OW, results were directionally consistent with the primary analysis. Robotic stapler use remained associated with lower air leak (11.29% vs. 15.25%), overall complications (20.57% vs. 26.33%), conversion (0.75% vs. 4.60%), and transfusion (1.25% vs. 1.77%), as well as shorter operative time (210 vs. 225 min) and LOS (4.0 vs. 5.4 days). Total hospital cost (USD 26,370.3 vs. 27,365.2) and total 30-day perioperative cost (USD 28,039.2 vs. 29,157.1) were also lower (Supplementary Tables 4a-4c).

### Subgroup analyses

Directionally similar associations were observed in the lobectomy and segmentectomy/wedge resection subgroups. In lobectomy, robotic stapler was associated with lower complication and conversion rates, shorter operative time, and reduced LOS (Supplementary Table 2a). Similar patterns were observed in segmentectomy/wedge resection subgroup (Supplementary Table 3a). Total hospital and 30-day perioperative costs were lower in the sublobar subgroup, whereas cost differences in the lobectomy subgroup were not statistically significant after weighting (Supplementary Tables 2b-3b).

### Risk factors for air leak and conversion

**Fig. 2:**
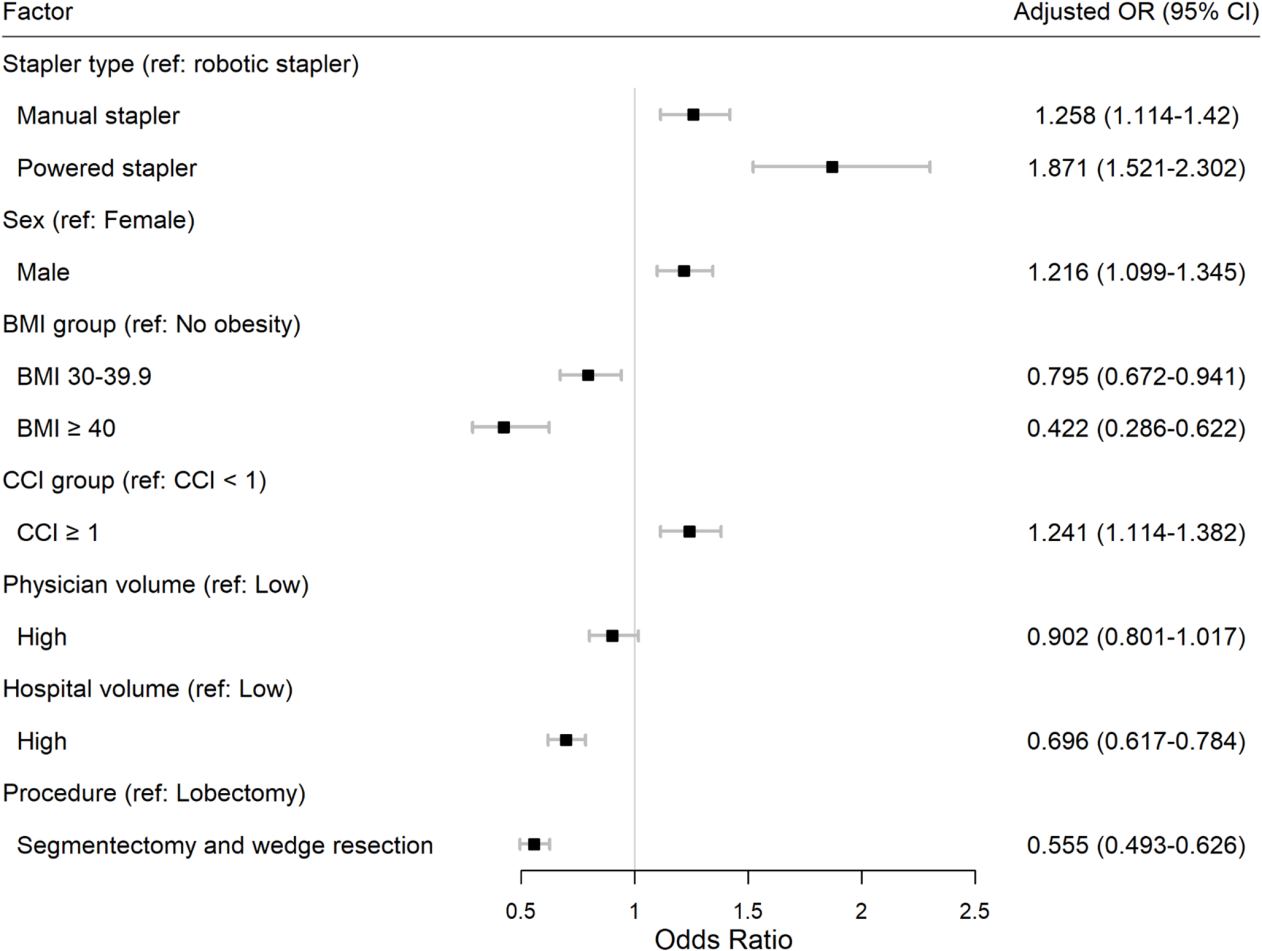
Factors associated with air leak. Odds ratio and 95% confidence interval (CI) were estimated using multivariable logistic regression after OW. *The cut-offs for physician volume and hospital volume were defined as their respective medians

**Fig. 3:**
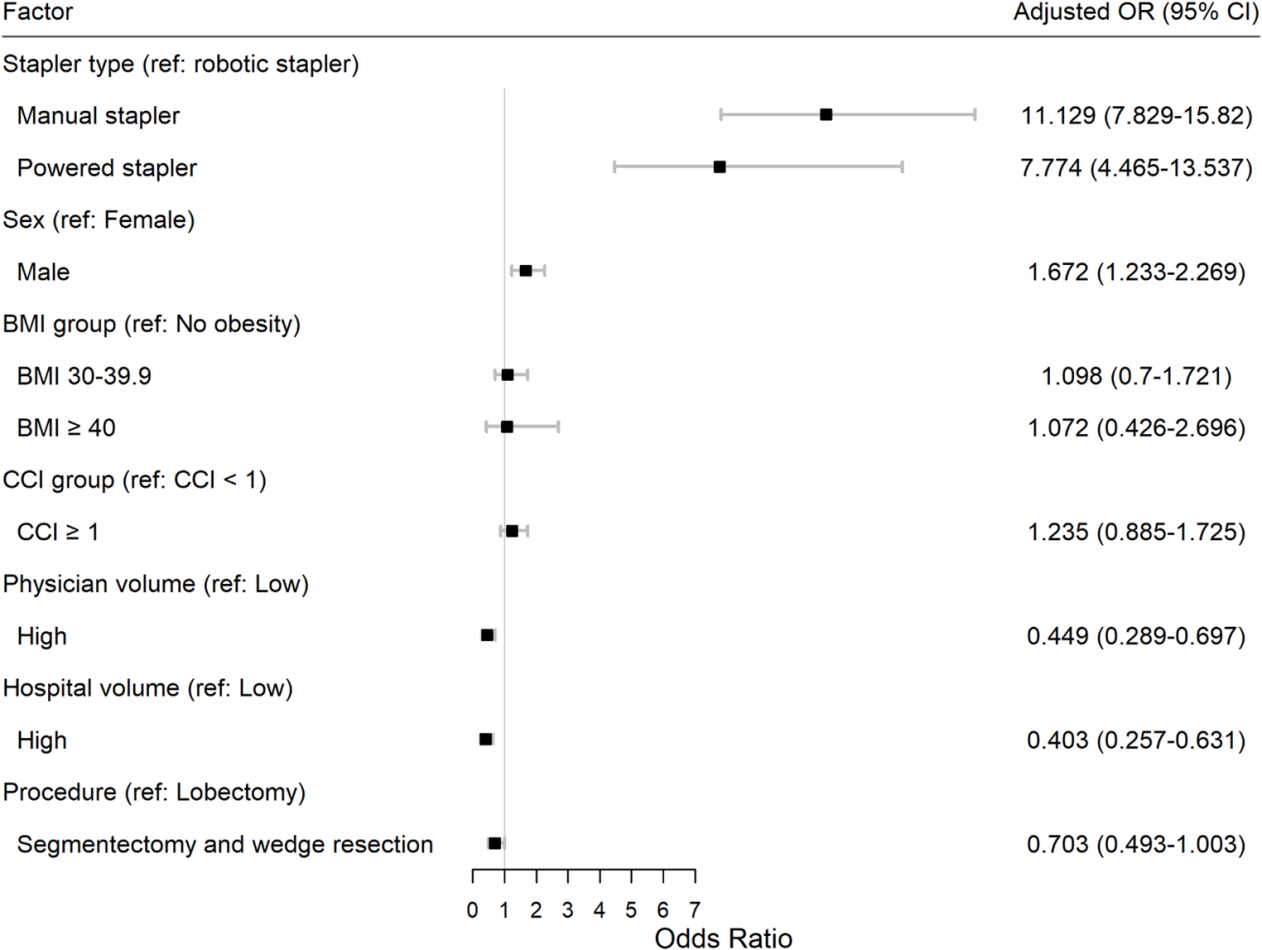
Factors associated with conversion. Odds ratio and 95% confidence interval (CI) were estimated using multivariable logistic regression after OW. *The cut-offs for physician volume and hospital volume were defined as their respective medians

Both manual (OR:1.258 [1.114–1.420]) and powered bedside staplers (OR: 1.871 [1.521–2.302]) were associated with a significantly higher risk of air leak compared with robotic staplers. Additional risk factors included male sex, absence of obesity, higher CCI, and lower hospital volume (Fig. 2). Differences were more pronounced for conversion: manual and powered staplers were associated with substantially higher odds of conversion (OR: 11.129 [7.829–15.82]; 7.774 [4.465–13.537]), respectively), along with male sex, low physician volume, and low hospital volume (Fig. 3).

## Discussion

To our knowledge, this is the largest study to evaluate clinical and economic outcomes associated with stapler type in robotic lung resections, incorporating the newest generation of stapling technologies. Robotic staplers were associated with fewer pulmonary complications, including air leak, pneumothorax, and pneumonia, along with lower transfusion and conversion rates, shorter operative time and LOS, and lower index and 30-day perioperative costs. Multivariable analyses consistently demonstrated higher risks of air leak and conversion with both manual and powered bedside staplers compared with robotic staplers, suggesting that stapler modality within RATS may be associated with differences in outcomes.

Robotic and bedside staplers differ in articulation, operator control, and workflow, but PHD does not capture staple formation, tissue compression, bedside assistant experience, number or type of staple loads, reasons for conversion, or intraoperative technical events. Accordingly, the mechanisms underlying the observed associations cannot be determined from these data. Differences may reflect stapler selection, surgeon and institutional experience, case complexity, perioperative care pathways, or other unmeasured factors in addition to stapler modality.

Our air leak findings align with Zervos et al. [11]. and help clarify the results reported by Phillips et al. [12], which relied on earlier-generation devices and smaller cohorts. However, the outcome was based on diagnosis and procedure coding during the index hospitalization and did not specifically identify prolonged air leak, the more clinically established endpoint. Both manual and powered bedside staplers were risk factors for air leak. In contrast to VATS-focused literature favoring powered staplers [24], powered bedside staplers demonstrated higher adjusted odds of air leak than manual staplers in our RATS cohort, possibly reflecting case selection, tactile feedback differences, or variability in assistant training but these associations should not be interpreted as proof of a device-specific mechanism. Other risk factors are consistent with prior studies [25–28].

Conversion is clinically important because it is associated with longer LOS, readmissions, and higher costs [18]. In this study, bedside stapler use was associated with higher conversion rates, including in the mix-used robotic sensitivity analysis. However, PHD does not identify the reason for conversion or whether conversion was related to stapling. The magnitude of the observed difference may reflect unmeasured case complexity, surgeon experience, institutional practice, or selection of stapler modality. Prior studies have reported differences in conversion between VATS and RATS at the platform level [18, 29–31], but those findings do not establish a stapler-specific causal effect.

Operative time was shorter with robotic staplers, whereas earlier studies reported no difference or longer times [11, 12]. Our later study window likely captured surgeons beyond early adoption phases, suggesting improved efficiency through reduced coordination delays and instrument exchanges.

Our cost findings were consistent with the clinical results. From the hospital perspective, despite robotic staplers having higher device specific costs, overall costs were lower, driven by reduced room-and-board, pharmacy and respiratory-therapy costs associated with shorter LOS, fewer conversions and complications. This suggests that higher upfront device costs may be partially offset by lower downstream cost, although formal cost-effectiveness was not evaluated. While prior robotic lobectomy studies reported no significant cost difference between stapler types [11], similar cost reductions have been reported in robotic colorectal procedures [32]. Interestingly, ICU utilization in the robotic stapler group was nearly half that of the handheld stapler group. This directly impacted the room & board costs, which were approximately 20% higher in the handheld group. Rationale for ICU use cannot be determined with this dataset however, and is known to be subjective and vary not only from hospital to hospital, but also surgeon to surgeon at the same site [33].

We extend prior work by including segmentectomy and wedge resection, which were absent from earlier stapler comparisons. Directionally similar associations were observed in the combined sublobar subgroup; however, ICD-10-PCS codes could not reliably distinguish segmentectomy from wedge resection. Because these procedures differ in technical complexity, operative duration, air leak risk, and stapler use, the subgroup findings should be interpreted cautiously [34–36].

Strengths include a large contemporary cohort, a prespecified published protocol, detailed chargemaster data, and use of propensity score overlap weighting. Several limitations are important. First, residual confounding remains likely because PHD does not capture tumor size, cancer stage, lobe or tumor location, fissure completeness, hilar complexity, surgeon robotic experience at the time of surgery, bedside assistant experience, ERAS pathways, number of staple firings, or reasons for conversion. Large pre-weighting differences in hospital and physician volume suggest that the groups arose from different practice environments. Second, OW estimates the ATO; therefore, the findings apply most directly to patients whose measured characteristics made either stapler strategy plausible and should not be assumed to generalize to patients at the extremes of the propensity score distribution. Although OW uses bounded weights and reduces instability from extreme weights relative to standard IPTW, it cannot address unmeasured confounding or limited overlap in unmeasured clinical characteristics. Third, the primary analysis excluded mixed-use cases, creating cleaner exposure groups but potentially reducing representativeness, although findings were consistent after including mixed-use cases. Fourth, coded air leak did not specifically identify prolonged air leak, and segmentectomy could not be separated from wedge resection. Finally, multiple secondary outcomes were assessed without multiplicity adjustment, and those findings should be considered exploratory.

## Conclusions

Robotic lung resection using robotic staplers were associated with fewer complications and conversions, shorter operative time and LOS, and lower index and 30-day costs. These findings were directionally consistent across lobectomy and sublobular resections. However, the findings should not be interpreted as evidence that stapler type alone caused the observed differences, and should be considered in light of potential residual confounding and heterogeneity in exposure .

## Supplementary Information

Below is the link to the electronic supplementary material.

Supplementary Material 1

## Data Availability

The data supporting these findings are available from Premier Inc. through the Premier Healthcare Database under license; the data are not publicly available from the authors.
