# Supplementary material for: Robotic vs. bedside staplers in robotic lung resection: a real-world cohort study
Source: J Robot Surg. 2026 Aug 6;20(1):797. doi: 10.1007/s11701-026-03772-0 (PMC13442317; doi:10.1007/s11701-026-03772-0)
Supplement: Supplementary file 1 [file 11701_2026_3772_MOESM1_ESM.docx]

**Supplementary Materials**

**S Table 1. ICD and CPT Codes for Robotic Lung Resection Identifications**

| **Code Type** | **Codes** | **Descriptions** |
| --- | --- | --- |
| Lung resection | | |
| CPT/HCPCS | 32480, 32482, 32486, 32663, 32668, 32670 | Lobectomy |
|  | 32505, 32666, 32484, 32669 | Segmentectomy and wedge resection |
| ICD-10-PCS | 0BTC0ZZ, 0BTC4ZZ, 0BTD0ZZ, 0BTD4ZZ, 0BTF0ZZ, 0BTF4ZZ, 0BTG0ZZ, 0BTG4ZZ, 0BTJ0ZZ, 0BTJ4ZZ | Lobectomy |
|  | 0BBC0ZX, 0BBC0ZZ, 0BBC4ZX, 0BBC4ZZ, 0BBD0ZX, 0BBD0ZZ,  0BBD4ZX, 0BBD4ZZ,  0BBF0ZX, 0BBF0ZZ,  0BBF4ZX, 0BBF4ZZ,  0BBG0ZX, 0BBG0ZZ,  0BBG4ZX, 0BBG4ZZ,  0BBH0ZX, 0BBH0ZZ,  0BBH4ZX, 0BBH4ZZ,  0BBJ0ZX, 0BBJ0ZZ,  0BBJ4ZX, 0BBJ4ZZ,  0BBK0ZX, 0BBK0ZZ,  0BBK4ZX, 0BBK4ZZ,  0BBL0ZX, 0BBL0ZZ,  0BBL4ZX, 0BBL4ZZ,  0BBM0ZX, 0BBM0ZZ,  0BBM4ZX, 0BBM4ZZ | Segmentectomy and wedge resection |
| Robotic procedure | | |
| CPT/HCPCS | S2900 | Surgical techniques requiring use of robotic surgical system |
| ICD-10-PCS | 8E0W0CZ, 8E0W3CZ, 8E0W4CZ, 8E0W7CZ, 8E0W8CZ, 8E0WXCZ | Robotic Assisted Procedure of Trunk Region |

**S Table 2a. Clinical Outcomes for Robotic Lobectomy by Stapler Types**

| *Clinical Outcomes* | *Summary statistics* | | *p-value* | *Summary statistics* | | *p-value* |
| --- | --- | --- | --- | --- | --- | --- |
|  | *Before weighting* | |  | *After weighting* | |  |
|  | **Robotic stapler** | **Bedside stapler** |  | **Robotic stapler** | **Bedside stapler** |  |
|  | **(N = 6,619)** | **(N = 2,589)** |  | **(N* = 4,312)** | **(N* = 1,713)** |  |
| ***Overall Complications*** | 2062 (31.15%) | 862 (33.29%) | 0.049 | 28.38% | 36.46% | < 0.001 |
| *Air Leak* | 1179 (17.81%) | 503 (19.43%) | 0.072 | 16.05% | 21.56% | < 0.001 |
| *Pneumothorax* | 878 (13.26%) | 393 (15.18%) | 0.019 | 12.08% | 16.41% | < 0.001 |
| *Bronchopleural Fistula* | 15 (0.23%) | 13 (0.5%) | 0.036 | 0.24% | 0.3% | 0.775 |
| *Bleeding* | 435 (6.57%) | 149 (5.76%) | 0.154 | 5.86% | 6.98% | 0.109 |
| *Acute Post Hemorrhagic Anemia* | 427 (6.45%) | 145 (5.6%) | 0.137 | 5.79% | 6.76% | 0.169 |
| *Pneumonia* | 150 (2.27%) | 75 (2.9%) | 0.084 | 1.84% | 3.57% | < 0.001 |
| *Infection* | 41 (0.62%) | 15 (0.58%) | 0.883 | 0.74% | 0.73% | 1.000 |
| ***Transfusion*** | 112 (1.69%) | 69 (2.67%) | 0.003 | 1.70% | 2.22% | 0.169 |
| ***Conversion*** | 84 (1.27%) | 112 (4.33%) | < 0.001 | 1.00% | 6.6% | < 0.001 |
| ***Operative Time:*** *Median (IQR)* | 240 (192-300) | 225 (183-285) | < 0.001 | 239 (182-297) | 255 (210-315) | < 0.001 |
| ***Length of Stay, LOS:*** *Mean (SD)* | 4.2 (4.7) | 4.9 (4.7) | < 0.001 | 4.2 (4.9) | 5.4 (5.1) | < 0.001 |
| ***ICU*** | 1368 (20.67%) | 1555 (60.06%) | < 0.001 | 20.18% | 42.39% | < 0.001 |

*Data presented as* ***frequency (%)****,* ***mean (SD)****, or* ***median (IQR)****.* ***N*****: Effective sample size after weighting*

**S Table 2b. Costs for Robotic Lobectomy by Stapler Types Before and After Overlap Weighting**

| *Costs items* | *Summary statistics* | | *p-value* | *Summary statistics* | | *p-value* |
| --- | --- | --- | --- | --- | --- | --- |
| *Mean (SD)* | *Before weighting* | |  | *After weighting* | |  |
|  | **Robotic stapler** | **Bedside stapler** |  | **Robotic stapler** | **Bedside stapler** |  |
|  | **(N = 6,619)** | **(N = 2,589)** |  | **(N* = 4,312)** | **(N* = 1,713)** |  |
| ***Total hospital costs*** | 28,704.7 (15,550.1) | 25,279.9 (14,234.7) | < 0.001 | 28,003.2 (15,147.7) | 28,545.9 (16,347.3) | 0.256 |
| *Stapler* | 2,859.3 (2,593.2) | 1,223.6 (1,157.7) | < 0.001 | 2,827.6 (2,592.7) | 1,345.9 (1,396.3) | < 0.001 |
| *Other Surgical Supply* | 3,925.7 (4,014.2) | 3,887.8 (2,798) | 0.608 | 3,957 (4,065.6) | 4,084.8 (3,084.5) | 0.184 |
| *Room and Board* | 6,485.6 (6,381) | 5,726.2 (6,425.7) | < 0.001 | 6,229 (6,094.8) | 7,473.1 (7,352.2) | < 0.001 |
| *Anesthesia* | 808.3 (846.1) | 733.7 (602.1) | < 0.001 | 731.2 (754.3) | 675.7 (711.6) | 0.009 |
| *Operating Room†* | 8,836.6 (5,239.5) | 7,803.7 (4,215.1) | < 0.001 | 8,432.1 (4,953.1) | 8,334 (4,632.2) | 0.477 |
| *Recovery Room* | 716.6 (581.7) | 618.6 (599.7) | < 0.001 | 733.1 (574.6) | 632.5 (684.3) | < 0.001 |
| *Pharmacy* | 1,166.8 (986) | 1,160.1 (1,099.6) | 0.787 | 1,168.5 (999.5) | 1,409.5 (1,203) | < 0.001 |
| *Respiratory Therapy* | 568.1 (1,857.6) | 587.8 (1,389.7) | 0.582 | 563.5 (1,655) | 727.5 (1,625.9) | < 0.001 |
| *Miscellaneous* | 2,776.7 (2,309.1) | 3,320.1 (2,493.9) | < 0.001 | 2,833.1 (2,249.6) | 3,483.3 (2,852.9) | < 0.001 |
| ***Total 30-day perioperative costs*** | 30,414.3 (17,708.7) | 27,054.7 (16,570.4) | < 0.001 | 29,805.2 (17,390.8) | 30,509 (18,814.3) | 0.199 |

*Data presented as* ***mean (SD)****.* ***N*****: Effective sample size after weighting*

*†Operating room cost excludes stapler cost.*

**S Table 3a. Clinical Outcomes for Robotic Segmentectomy/Wedge Resection by Stapler Types.**

| *Clinical Outcomes* | *Summary statistics* | | *p-value* | *Summary statistics* | | *p-value* |
| --- | --- | --- | --- | --- | --- | --- |
|  | *Before weighting* | |  | *After weighting* | |  |
|  | **Robotic stapler** | **Bedside stapler** |  | **Robotic stapler** | **Bedside stapler** |  |
|  | **(N = 5,510)** | **(N = 1,680)** |  | **(N* = 4,263)** | **(N* = 1,334)** |  |
| ***Overall Complications*** | 725 (13.16%) | 198 (11.79%) | 0.145 | 457 (10.72%) | 180.2 (13.51%) | 0.007 |
| *Air Leak* | 352 (6.39%) | 113 (6.73%) | 0.611 | 223.2 (5.24%) | 99.1 (7.43%) | 0.004 |
| *Pneumothorax* | 345 (6.26%) | 72 (4.29%) | 0.002 | 213.1 (5%) | 68.4 (5.13%) | 0.886 |
| *Bronchopleural Fistula* | 4 (0.07%) | 4 (0.24%) | 0.092 | 2.9 (0.07%) | 1.8 (0.13%) | 0.343 |
| *Bleeding* | 140 (2.54%) | 32 (1.9%) | 0.145 | 89 (2.09%) | 28.3 (2.12%) | 1 |
| *Acute Post Hemorrhagic Anemia* | 137 (2.49%) | 30 (1.79%) | 0.097 | 88.1 (2.07%) | 26.7 (2%) | 1 |
| *Pneumonia* | 43 (0.78%) | 23 (1.37%) | 0.039 | 27 (0.63%) | 25 (1.87%) | < 0.001 |
| *Infection* | 12 (0.22%) | 4 (0.24%) | 0.775 | 6 (0.14%) | 4.8 (0.36%) | 0.146 |
| ***Transfusion*** | 47 (0.85%) | 14 (0.83%) | 1 | 31.1 (0.73%) | 14.7 (1.1%) | 0.166 |
| ***Conversion*** | 27 (0.49%) | 27 (1.61%) | < 0.001 | 18.2 (0.43%) | 30.3 (2.27%) | < 0.001 |
| ***Operative Time:*** *Median (IQR)* | 180 (141-240) | 180 (146-240) | 0.142 | 180 (140-240) | 195 (150-270) | < 0.001 |
| ***Length of Stay, LOS:*** *Mean (SD)* | 3.6 (4.5) | 4.9 (6.5) | < 0.001 | 3.7 (5.1) | 5.3 (6.7) | < 0.001 |
| ***ICU*** | 998 (18.11%) | 785 (46.73%) | < 0.001 | 18.41% | 33.79% | < 0.001 |

*Data presented as* ***frequency (%)****,* ***mean (SD)****, or* ***median (IQR)****.* ***N*****: Effective sample size after weighting*

**S Table 3b. Costs for Robotic Segmentectomy/Wedge by Stapler Types Before and After Overlap Weighting**

| *Costs items* | *Summary statistics* | | *p-value* | *Summary statistics* | | *p-value* |
| --- | --- | --- | --- | --- | --- | --- |
| *Mean (SD)* | *Before weighting* | |  | *After weighting* | |  |
|  | **Robotic stapler** | **Bedside stapler** |  | **Robotic stapler** | **Bedside stapler** |  |
|  | **(N = 5,510)** | **(N = 1,680)** |  | **(N* = 4,263)** | **(N* = 1,334)** |  |
| ***Total hospital costs*** | 24,199.9 (14,165.2) | 23,915.6 (16,146.5) | 0.516 | 24,253 (14,644.3) | 25,951.6 (17,375.7) | 0.002 |
| *Stapler* | 2,371.8 (2,288.8) | 1,279.4 (1,447.2) | < 0.001 | 2,310.4 (2,228.8) | 1,429.7 (1,645.8) | < 0.001 |
| *Other Surgical Supply* | 3,386.6 (3,386.7) | 3,560.7 (3,204.7) | 0.054 | 3,428.1 (3,434.9) | 3,725.6 (3,433.5) | 0.007 |
| *Room and Board* | 5,467.4 (6,535.5) | 5,819.2 (7,379.7) | 0.079 | 5,504.5 (6,801.7) | 6,900.1 (7,989.4) | < 0.001 |
| *Anesthesia* | 673.5 (657.9) | 691.1 (587) | 0.296 | 650 (628.8) | 675.8 (659.8) | 0.217 |
| *Operating Room†* | 7,192.9 (4,036.7) | 6,802.6 (4,140.8) | < 0.001 | 7,100.8 (3,987.1) | 7,122.3 (4,478) | 0.878 |
| *Recovery Room* | 708.8 (545.2) | 668.1 (585.2) | 0.011 | 719.6 (550.8) | 693.4 (632.3) | 0.176 |
| *Pharmacy* | 1,108.8 (1,029.1) | 1,170 (1,317.7) | 0.081 | 1,145.7 (1,098) | 1,299.6 (1,379.4) | < 0.001 |
| *Respiratory Therapy* | 383.8 (769.4) | 507 (1,014.1) | < 0.001 | 475.1 (1,683.8) | 682.7 (1,991.6) | < 0.001 |
| *Miscellaneous* | 2,422.1 (2,192.6) | 3,180.5 (3,231.7) | < 0.001 | 2,516.4 (2,347.1) | 3,247.7 (3,436.3) | < 0.001 |
| ***Total 30-day perioperative costs*** | 25,726 (15,966.8) | 25,584.8 (18,260) | 0.775 | 25,778.4 (16,515.3) | 27,540.7 (19,627.8) | 0.004 |

*Data presented as* ***mean (SD)****.* ***N*****: Effective sample size after weighting*

*†Operating room cost excludes stapler cost.*

**S Table 4a. Baseline Characteristics by Stapler Type in Robotic Lung Resection Before and After Overlap Weighting (Mixed-Use Cases)**

| *Characteristics* | *Summary statistics* | | *p-value* | *ASMD* | *Summary statistics* | | *p-value* | *ASMD* |
| --- | --- | --- | --- | --- | --- | --- | --- | --- |
|  | *Before weighting* | |  |  | *After weighting* | |  |  |
|  | **Robotic stapler** | **Bedside stapler** |  |  | **Robotic stapler** | **Bedside stapler** |  |  |
|  | **(N = 12,129)** | **(N = 4,269)** |  |  | **(N* = 8,637)** | **(N* = 3,071)** |  |  |
| ***Age:*** *Mean (SD)* | 66.38 (10.9) | 67.5 (11.7) | <0.001 | 0.0991 | 66 (11.17) | 66 (11.85) | 1 | < 0.001 |
| ***Gender*** |  |  | <0.001 |  |  |  | 1 |  |
| *Female* | 6810 (56.15%) | 2250 (52.71%) |  | 0.0691 | 53.75% | 53.73% |  | < 0.001 |
| *Male* | 5319 (43.85%) | 2019 (47.29%) |  | 0.0691 | 46.26% | 46.24% |  | < 0.001 |
| ***Race Group*** |  |  | <0.001 |  |  |  | 1 |  |
| *Black* | 1128 (9.3%) | 503 (11.78%) |  | 0.0809 | 12.51% | 12.50% |  | < 0.001 |
| *Other/unknown* | 1367 (11.27%) | 433 (10.14%) |  | 0.0365 | 10.61% | 10.62% |  | < 0.001 |
| *White* | 9634 (79.43%) | 3333 (78.07%) |  | 0.0331 | 76.89% | 76.88% |  | < 0.001 |
| ***Payor*** |  |  | <0.001 |  |  |  | 1 |  |
| *Commercial* | 3226 (26.6%) | 948 (22.21%) |  | 0.1024 | 26.57% | 26.57% |  | < 0.001 |
| *Medicaid* | 807 (6.65%) | 270 (6.32%) |  | 0.0133 | 7.00% | 7.00% |  | < 0.001 |
| *Medicare* | 7559 (62.32%) | 2934 (68.73%) |  | 0.1351 | 62.74% | 62.72% |  | < 0.001 |
| *Other* | 537 (4.43%) | 117 (2.74%) |  | 0.0908 | 3.71% | 3.71% |  | < 0.001 |
| ***COPD/emphysema*** |  |  | 0.158 |  |  |  | 1 |  |
| *NO* | 7325 (60.39%) | 2525 (59.15%) |  | 0.0254 | 59.39% | 59.39% |  | < 0.001 |
| *YES* | 4804 (39.61%) | 1744 (40.85%) |  | 0.0254 | 40.61% | 40.61% |  | < 0.001 |
| ***CCI*** |  |  | <0.001 |  |  |  | 1 |  |
| *CCI < 1* | 4490 (37.02%) | 1408 (32.98%) |  | 0.0847 | 34.46% | 34.45% |  | < 0.001 |
| *CCI ≥ 1* | 7639 (62.98%) | 2861 (67.02%) |  | 0.0847 | 65.55% | 65.55% |  | < 0.001 |
| ***Obesity*** |  |  | <0.001 |  |  |  | 1 |  |
| *No obesity* | 10210 (84.18%) | 3723 (87.21%) |  | 0.0867 | 85.33% | 85.31% |  | < 0.001 |
| *BMI 30-39.9* | 1410 (11.63%) | 413 (9.67%) |  | 0.0633 | 10.88% | 10.88% |  | < 0.001 |
| *BMI ≥ 40* | 509 (4.2%) | 133 (3.12%) |  | 0.0576 | 3.79% | 3.78% |  | < 0.001 |
| ***Teaching Hospital*** |  |  | <0.001 |  |  |  | 1 |  |
| *NO* | 4297 (35.43%) | 433 (10.14%) |  | 0.6322 | 17.45% | 17.45% |  | < 0.001 |
| *YES* | 7832 (64.57%) | 3836 (89.86%) |  | 0.6322 | 82.55% | 82.55% |  | < 0.001 |
| ***Hospital Bed Size*** |  |  | <0.001 |  |  |  | 1 |  |
| *<300* | 2178 (17.96%) | 443 (10.38%) |  | 0.2187 | 13.31% | 13.32% |  | < 0.001 |
| *300-399* | 1109 (9.14%) | 406 (9.51%) |  | 0.0126 | 10.74% | 10.75% |  | < 0.001 |
| *400-499* | 2028 (16.72%) | 262 (6.14%) |  | 0.3373 | 10.54% | 10.55% |  | < 0.001 |
| *≥500* | 6814 (56.18%) | 3158 (73.98%) |  | 0.38 | 65.42% | 65.39% |  | < 0.001 |
| ***Urban/Rural*** |  |  | <0.001 |  |  |  | 1 |  |
| *Rural* | 466 (3.84%) | 30 (0.7%) |  | 0.2118 | 1.30% | 1.30% |  | < 0.001 |
| *Urban* | 11663 (96.16%) | 4239 (99.3%) |  | 0.2118 | 98.70% | 98.70% |  | < 0.001 |
| ***Performed by Specialist*** |  |  | 0.004 |  |  |  | 1.000 |  |
| *NO* | 4753 (39.19%) | 1566 (36.68%) |  | 0.0516 | 43.23% | 43.21% |  | < 0.001 |
| *YES* | 7376 (60.81%) | 2703 (63.32%) |  | 0.0516 | 56.77% | 56.76% |  | < 0.001 |
| ***Hospital Volume:*** *Mean (SD)* | 398.37 (310.35) | 956 (858.4) | <0.001 | 0.864 | 489.85 (333.91) | 489.85 (525.77) | 1 | < 0.001 |
| ***Physician Volume:*** *Mean (SD)* | 198.51 (189.09) | 594.3 (651.74) | <0.001 | 0.8248 | 229.76 (190.25) | 229.76 (260.76) | 1 | < 0.001 |
| ***Procedure Name*** |  |  | <0.001 |  |  |  | 1 |  |
| *Lobectomy* | 6619 (54.57%) | 2589 (60.65%) |  | 0.1232 | 55.87% | 55.85% |  | < 0.001 |
| *Segmentectomy/wedge resection* | 5510 (45.43%) | 1680 (39.35%) |  | 0.1232 | 44.14% | 44.12% |  | < 0.001 |
| ***Malignant Cancer*** |  |  | <0.001 |  |  |  | 1 |  |
| *NO* | 3761 (31.01%) | 1515 (35.49%) |  | 0.0952 | 33.5% | 33.51% |  | < 0.001 |
| *YES* | 8368 (68.99%) | 2754 (64.51%) |  | 0.0952 | 66.5% | 66.49% |  | < 0.001 |

***ASMD:*** *absolute standardized mean difference;* ***N*****: Effective sample size after weighting;* ***SD:*** *standard deviation;* ***BMI:*** *body mass index;* ***CCI:*** *Charlson comorbidity index*

**S Table 4b. Clinical Outcomes for Robotic Lung Resection by Stapler Types Before and After Overlap Weighting (Mixed-Use Cases)**

| *Clinical Outcomes* | *Summary statistics* | | *p-value* | *Summary statistics* | | *p-value* |
| --- | --- | --- | --- | --- | --- | --- |
|  | *Before weighting* | |  | *After weighting* | |  |
|  | **Robotic stapler** | **Bedside stapler** |  | **Robotic stapler** | **Bedside stapler** |  |
|  | **(N = 12,129)** | **(N = 4,269)** |  | **(N* = 8,637)** | **(N* = 3,071)** |  |
| ***Overall Complications*** | 2787 (22.98%) | 1060 (24.83%) | 0.015 | 20.57% | 26.33% | < 0.001 |
| *Air Leak* | 1531 (12.62%) | 616 (14.43%) | 0.003 | 11.29% | 15.25% | < 0.001 |
| *Pneumothorax* | 1223 (10.08%) | 465 (10.89%) | 0.135 | 8.99% | 11.47% | < 0.001 |
| *Bronchopleural Fistula* | 19 (0.16%) | 17 (0.4%) | 0.007 | 0.16% | 0.23% | 0.46 |
| *Bleeding* | 575 (4.74%) | 181 (4.24%) | 0.188 | 4.17% | 4.82% | 0.135 |
| *Acute Post Hemorrhagic Anemia* | 564 (4.65%) | 175 (4.1%) | 0.145 | 4.12% | 4.65% | 0.212 |
| *Pneumonia* | 193 (1.59%) | 98 (2.3%) | 0.004 | 1.33% | 2.83% | < 0.001 |
| *Infection* | 53 (0.44%) | 19 (0.45%) | 1 | 0.46% | 0.58% | 0.366 |
| ***Transfusion*** | 159 (1.31%) | 83 (1.94%) | 0.004 | 1.25% | 1.77% | 0.047 |
| ***Conversion*** | 111 (0.92%) | 139 (3.26%) | < 0.001 | 0.75% | 4.6% | < 0.001 |
| ***Operative Time:*** *Median (IQR)* | 212 (161-273) | 206 (157-270) | 0.974 | 210 (150-270) | 225 (165-295) | < 0.001 |
| ***Length of Stay, LOS:*** *Mean (SD)* | 3.9 (4.6) | 4.9 (5.5) | < 0.001 | 4 (5.1) | 5.4 (5.9) | < 0.001 |
| ***ICU*** | 2366 (19.51%) | 2340 (54.81%) | < 0.001 | 19.34% | 38.63% | < 0.001 |

*Data presented as* ***frequency (%)****,* ***mean (SD)****, or* ***median (IQR)****.* ***N*****: Effective sample size after weighting*

**S Table 4c. Costs for Robotic Lung Resection by Stapler Types Before and After Overlap Weighting (Mixed-Use Cases)**

| *Costs items* | *Summary statistics* | | *p-value* | *Summary statistics* | | *p-value* |
| --- | --- | --- | --- | --- | --- | --- |
| *Mean (SD)* | *Before weighting* | |  | *After weighting* | |  |
|  | **Robotic stapler** | **Bedside stapler** |  | **Robotic stapler** | **Bedside stapler** |  |
|  | **(N = 12,129)** | **(N = 4,269)** |  | **(N* = 8,637)** | **(N* = 3,071)** |  |
| ***Total hospital costs*** | 26,658.2 (15,103.8) | 24,743 (15,029.1) | < 0.001 | 26,370.3 (15,135.2) | 27,365.2 (16,839.6) | 0.006 |
| *Stapler* | 2,637.9 (2,471.5) | 1,245.6 (1,279.6) | < 0.001 | 2,585.1 (2,442.9) | 1,387.3 (1,510.5) | < 0.001 |
| *Other Surgical Supply* | 3,680.8 (3,751.7) | 3,759.1 (2,968.6) | 0.168 | 3,723.3 (3,809.4) | 3,922.5 (3,244.5) | 0.006 |
| *Room and Board* | 6,023.1 (6,471.3) | 5,762.8 (6,816.4) | 0.03 | 5,915.9 (6,473.3) | 7,221.8 (7,658.3) | < 0.001 |
| *Anesthesia* | 747.1 (769.3) | 717 (596.5) | 0.009 | 696.4 (703.7) | 676.3 (689.9) | 0.178 |
| *Operating Room†* | 8,089.9 (4,801.3) | 7,409.7 (4,214) | < 0.001 | 7,863.3 (4,627.8) | 7,783.8 (4,599.6) | 0.423 |
| *Recovery Room* | 713 (565.4) | 638.1 (594.4) | < 0.001 | 727.5 (564) | 660.8 (663.2) | < 0.001 |
| *Pharmacy* | 1,140.4 (1,006.2) | 1,164 (1,190.1) | 0.248 | 1,163.4 (1,054) | 1,357.3 (1,284.4) | < 0.001 |
| *Respiratory Therapy* | 510.5 (1,690.1) | 598.2 (1,601) | 0.002 | 532 (1,763.6) | 704.8 (1,798) | < 0.001 |
| *Miscellaneous* | 2,615.6 (2,263.7) | 3,265.1 (2,807.9) | < 0.001 | 2,703 (2,326.8) | 3,369 (3,120) | < 0.001 |
| ***Total 30-day perioperative costs*** | 28,284.5 (17,099) | 26,476 (17,268.2) | < 0.001 | 28,039.2 (17,194.4) | 29,157.1 (19,214.5) | 0.006 |

*Data presented as* ***mean (SD)****.* ***N*****: Effective sample size after weighting*

*†Operating room cost excludes stapler cost.*
